# Decades of failure to prevent harm to patients—where are we going wrong? A mixed methods study of the perspectives of health services staff across Australia and internationally

**DOI:** 10.3389/frhs.2025.1645575

**Published:** 2025-09-02

**Authors:** Mia Bierbaum, Yinghua Yu, Charlotte J. Molloy, Lorelle Bowditch, Paul M. Salmon, Sandy Middleton, Jeffrey Braithwaite, Peter Hibbert

**Affiliations:** ^1^ Australian Institute of Health Innovation (AIHI), Macquarie University, Sydney, NSW, Australia; ^2^ IIMPACT in Health, Allied Health and Human Performance, University of South Australia, Adelaide, SA, Australia; ^3^ Centre for Human Factors and Sociotechnical Systems, University of the Sunshine Coast, Sunshine Coast, QLD, Australia; ^4^ Nursing Research Institute, Australian Catholic University, Sydney, NSW, Australia

**Keywords:** patient safety, hospital incident reporting, risk management, hospitals, medical errors, incident review, human factors, adverse events

## Abstract

**Context:**

Patient Safety Incident (PSI) reviews are undertaken frequently across health services in response to serious adverse events. This study investigated experiences and perceptions of healthcare professionals involved in incident review processes across four jurisdictions in Australia, alongside insights from international patient safety experts. These findings will inform the co-design of improvements to PSI reviews.

**Methods:**

Semi-structured interviews and focus groups were conducted, and participants completed an attitudinal and demographic survey. Inductive thematic analysis was conducted, and findings were deductively mapped against the Consolidated Framework for Implementation Research.

**Findings:**

Australian (*n* = 99) and international (*n* = 11) participants took part in one of 25 focus groups (*n* = 78) or 32 interviews. Most participants (*n* = 99) completed the survey. Nearly all survey participants agreed/strongly agreed that PSI reviews are valuable for improving patient safety (95%), particularly when human factors and contextual influences on performance (76%) are considered. Two-thirds of participants agreed that investigations help prevent PSI recurrence (68%), avoid unfair blame (67%), and support continuous improvement (61%). However, fewer participants felt recommendations are consistently accepted by organisations (58%) or are appropriately targeted within the healthcare system (57%). Key strengths and challenges of the PSI review process were identified across three themes**:** Selection of PSI Reviews; Reviews, Recommendations and Implementation; and Health Organisations and Wider System Influences. Key PSI review challenges included: limited capacity and engagement, high staff workloads, turnover, and burnout, as well as variable skills, and limited human factors and systems thinking experience across review teams. Despite strong efforts to reduce a punitive culture, resistance to reporting and blame persists across some hospitals. Participants highlighted a learned powerlessness when developing systems thinking-based recommendations, resulting in the development of weaker, less resource intensive recommendations. Limited sharing of learnings and feedback on review findings, and variable monitoring, evaluation and accountability of recommendation implementation were also common challenges.

**Conclusions:**

These findings have identified a need for system re-engineering of PSI reviews to address identified challenges and will inform the development of Best Practice Principles and a codesign of patient safety tactics for trial in-situ.

## Introduction

Healthcare systems are designed to care for patients, however inadvertent harm can often occur (1–4). Patient safety incidents (PSIs) that arise during the provision of healthcare, including unexpected death or serious harm, are a concern internationally and significantly contribute to patient morbidity and mortality (5). Internationally, 12% of patients receiving medical care experience harm (5), a third of which are deemed preventable (6), with patient harm contributing to over 2.6 million deaths annually in low to middle income countries (LMICs) alone (7, 8). The most common PSIs involve drug-related incidents (e.g., wrong drug or dose), surgical or procedural events (e.g., item retained during surgery, wrong-site surgery), patient care events (e.g., falls), and hospital acquired infections (6). Annually, PSIs are estimated to cost the US health system $17.1 billion (2008 figures) (9), and internationally PSIs have been estimated to cost 13% of healthcare budgets in developed countries (10). The estimated cost of PSIs in LMICs (including lost productivity), is estimated to be USD1.6 trillion annually (8). PSIs increase the burden for patients, staff and the health system through extended lengths of stay, preventable readmissions, increased demand for health service use, and overall poorer health outcomes (11, 12).

When a patient is harmed, healthcare organisations should report, manage and investigate the incident (13, 14), provide feedback to patients and families about review findings (11), and implement interventions designed to prevent future similar occurrences. The purpose of these incident reviews is to understand what happened and why, and to identify interventions to reduce the frequency of harm to other patients within the health system. In many countries this process is guided by incident best practice guidelines developed by health care organisations or national or state jurisdictions (15–19). By way of example, there are approximately 1,700 incident reviews of serious adverse events undertaken in Australian public health services each year (20). Reliable collated data from the US on these figures is not available. Conducting PSI reviews is resource and time intensive, involving multiple steps, and methodologies (15, 17, 18, 21–34) often with senior staff members (1, 15) (Box 1).

Box 1 **Ideal steps of a PSI review**

**Table:** 

Stages	Task
Initial response	The initial steps include the identification and reporting of the incident, followed by responding to imminent risk (15)
Severity assessment	Next, the level of harm of the incident is verified. The level of harm, or severity of an adverse event is assessed (15) and scored using various ratings depending on the jurisdiction (e.g., An Incident Severity Rating (ISR), (15) a Severity Assessment Code (SAC), (16) or Harm Score (11)). While they vary, these scores include similar levels of harm. For example, a Clinical Harm Score 1 refers to an unexpected death or Australian Sentinel Event, a Harm Score 2 refers to major harm, Harm Score 3 refers to minor harm, and Harm Score 4 refers to no harm or a near miss. (17) This rating is used to guide the method and depth of investigation and response to the incident. (15–17)
Methodology	A formal review process is then followed, particularly for serious harm incidents. There are a variety of incident analysis methods and models used, depending on the jurisdiction, incident, and resources available. Common recommended methods include Root Cause Analysis (RCA), (21) Root Cause Analysis and Action (RCA2), (22) Serious Adverse Event Review (SAER), (17) Accident Mapping Approach (AcciMap), (23, 24) London Protocol, (25) Failure Mode and Effects Analysis (FMEA), (26) Human Error and Patient Safety framework (HEAPs), (27) Systems Engineering Initiative for Patient Safety (SEIPS), (28) Systems Theoretic Accident Modelling and Processes (STAMP), (23, 29) or an aggregate review depending on the level of harm. (15) Other notable recommended methods include Functional Resonance Analysis Method (FRAM), (30, 31) The Events and Causal Factors (ECF), (32) The human factors analysis and classification system-healthcare (HFACs-healthcare), (33, 141) The Patient Safety Incident Response Framework (PSIRF), (18) and Hierarchical Task Analysis (HTA). (34)
Team	A review team is established - ideally including a review chair; a facilitator who is a quality and safety team member; review members who were not directly involved in the incident, but have representative clinical knowledge; an external team member who is a subject matter expert from outside of the organisation; and a consumer representative. (15)
Review	The team conduct the investigation processes (gathering data via document review, interviews and observations, establishing a timeline of events, conducting data analysis, identifying contributory factors, contextual influences, and incident outcomes) and produce a written report of findings and Specific, Measurable, Achievable, Relevant and Time-bound (SMART) recommendations, ideally with an implementation plan embedded. (15)
Report	The findings and recommendations are then reviewed and endorsed by an executive sponsor accountable for the review. (15)
Open disclosure	During this process, open disclosure is conducted with patients and families, including sharing the final report, findings, and action plan, as well as an apology. (15)
Implementation/monitoring	The recommendations are (ideally) implemented, and the progress and impact of this process is monitored. (15)
Sharing	Relevant authorities are notified of the review and findings. (15) Finally, findings are (ideally) shared with the broader health service. (15)

Sources: as shown; Authors’ conceptualisation.

Major reports in the UK and US at the turn of century were published highlighting the problem of patient safety (35, 36). Despite concerted efforts by policy-makers including the World Health Organisation, national and state-wide bodies, healthcare executives, clinicians, and patients, the evidence is clear that current incident review processes do not effectively reduce the frequency of PSIs causing preventable harm to patients (1, 37–40). Despite decades of research and vast resources invested in the review and response process, limited improvements have been observed in the rates of PSIs, in Australian hospitals (4, 11, 12), in the US (4, 6), and internationally (4, 41–45).

Healthcare systems are notoriously multifaceted and intricate: most frequently described as complex adaptive systems (46), and PSIs are grounded in these complexities, within a multitude of healthcare system conditions, events and failures (11). There are many known inter-dependent determinants that impact the effectiveness of a response to PSIs across multiple levels of the healthcare system, including the clinical microsystem, complicated funding arrangements, organisational features, and the density of the policy and regulation milieu. This complexity underlies the importance of examining this topic in such detail. These include a historical culture of blame and fear of punitive consequences (47, 48), poor reporting systems and processes (47, 48), labyrinth-like governance structures, workforce demands, capacity and capability, including the composition and skills of review teams, and the many types of investigation methods utilised (47, 49). Despite decades of PSI Reviews in healthcare settings internationally, persisting themes include a lack of feedback on review findings, a failure to identify contributory factors in the broader healthcare system, poor quality recommendations, and poor-to-no evidence-based implementation strategies (47, 48), with a lack of systematic follow-up and limited perceived improvements following the review and reporting process (48). Limited incorporation of implementation science theory and methodology as well as quality improvement methods may contribute to this poor implementation process (50–52). A strong safety culture, staff engagement, national safety policies, and regulatory bodies, as well as support and staff upskilling, shared learning and patient engagement, are all considered important facilitators of better PSI reviews and safer care (53).

PSI Review recommendations are often unspecific and lacking measurability (54), limiting their effectiveness (45); weak recommendations frequently focus on staff and individual factors, the sharp-end of healthcare—rather than addressing system-wide improvements (15, 55). Implementation of such recommendations (38, 54, 56) also has limited effectiveness on rates of adverse events (38, 56). Further evidence is needed to understand the determinants of effective development and implementation of recommendations resulting from PSI reviews, to see reductions in patient harm (1, 37–40).

Recent development in patient safety emphasises a significant shift from reactive, individual-blame-focused investigations towards more proactive, system-oriented approaches that prioritise learning and improvement (15, 17, 57). Frameworks such as the United Kingdom's Patient Safety Incident Response Framework (PSIRF) (18) and the new version of the London Protocol (58, 59) exemplify this transition, promoting a proportionate, risk-based response to incidents and embedding principles of openness, system learning (60), and patient and family engagement in the investigation process. Complementing this, emerging international models increasingly draw on restorative justice principles, which seek to address not only what happened, but also who was affected, how they were affected, and what is needed for healing and collective accountability (61, 62). A recent cross-country analysis of restorative initiatives in five countries further illustrates the potential of these approaches to reorient investigations toward dialogue, dignity and trust (61). These shifts align well with global priorities articulated in the World Health Organisation's Global Patient Safety Action Plan 2021-2030 (8), which calls for strengthening system-level responses and engaging affected parties as active partners in safety (63, 64). These redevelopments underpin the growing recognition that meaningful and compassionate investigation processes are essential not only for identifying systemic risks but also for addressing the broader emotional, relational and ethical dimensions of harm, including compounded harm (65, 66). In particular, we note that compounded harm is not limited to patients and families (67, 68): health professionals (including investigators and clinicians) (69) and consumer representatives (70) can also experience moral distress and emotional exhaustion. These harms highlight the need for systems that attend not only to technical processes but also to the emotional dimensions of safety reviews.

Although the PSI response challenges outlined above are well known, what is less well understood are the factors that are contributing to these issues. This study aims to understand what is and is not working, when, and why in relation to incident response. This will be achieved by examining the perceptions, experiences, and current practices in incident response, management, investigation, analysis and implementation of improvements, across a cohort of Australian health service staff and international patient safety experts. This study examines the entire lifecycle of incident response, as due to their complexity and interdependency, they may all impact a health care organisation's learning and action to prevent similar incidents occurring again.

The findings from this study will inform the development of Best Practice Principles, a co-design process to re-engineer patient safety tactics to reduce patient risk, and *in situ* feasibility testing of identified practice improvements. This codesign and testing process will strive to support healthcare organisations to improve and measure their response analysis and learning from incidents, thereby reducing the risk of preventable harm to patients.

## Methods

### Context

This study is funded by a National Health and Medical Research Council (NHMRC) Partnership Grant project aiming to investigate ways to improve the health systems' responses when patients are harmed across four Australian state and territory public health systems: New South Wales (NSW), Victoria (VIC), the Australian Capital Territory (ACT), and Queensland (QLD). Approximately 77% (20.0 million) of the Australian population reside, and 78% (5.3 million) of hospital admissions occur in these states (71).

Our broader project included participants with lived experience of harm, such as consumer advocates or consumer representatives (CRs), but their data are not included in this paper. Their perceptions and experiences on PSI review teams were already published (70). Two additional papers are currently in draft: one focuses on best practice principles for working with CRs, and another explores consumer needs and expectations at various stages of PSI reviews.

### Study type

This mixed methods research (MMR) study used a concurrent QUAL+QUAN design (72) to collect quantitative data from surveys and qualitative data from focus groups and interviews, with each dataset equally prioritised and findings compared and merged. This is aimed at increasing the validity of the data by exploring the survey data in more depth throughout the interviews and focus groups (73). The study is underpinned by a pragmatic worldview with the belief that utilising both data types will provide a more robust understanding of the key concepts (74). The reporting of this study was guided by the Good Reporting of A Mixed Methods Study (GRAMMS) checklist (75) and the Consolidated Criteria for REporting Qualitative (COREQ) guidance (76) (See Supplementary Files S1 and S2).

### Recruitment

Health service staff involved in PSI reviews were recruited through stratified convenience sampling (77) conducted by health services across four Australian states and territories (NSW, VIC, QLD, ACT), as well as key organisations involved in PSI responses who are partners on this grant, and chief investigators (CIs). This sampling method was used to ensure representation of staff in Australian public health systems who: undertake incident management and PSI reviews; co-commission and are “customers” of reviews e.g., clinical directors, who co-commission, undertake, and quality assure investigations e.g., safety and quality directors and staff; or are from statewide organisations involved in policy or innovation around PSI reviews. Key contacts sent recruitment invitations to potential participants, who then contacted researchers to express interest in participating in an interview or focus group, and to complete a survey. There was no direct recruitment by the researchers.

In addition, international patient safety experts (based on their recognised contributions to patient safety research, policy development, systems design or healthcare quality improvement at a national or international level) were recruited by the network of CIs and partner organisations affiliated with this grant. This involved sending out invitation emails to a stratified convenience sample based on involvement in PSI review processes, including senior staff (such as human factors specialists, policy advisors, academics, and healthcare leaders) from regulatory bodies, government and policy making organisations, such as England's Health Services Safety Investigations Body. There were some existing professional relationships between CIs and participants (due to the nature of working in the same research area/industry), however, participation was voluntary. Interested individuals responded by contacting the research team to complete informed consent documentation and schedule an interview.

### Data collection

#### Quantitative data

Once participants completed informed written consent, they completed an electronic demographic and attitudinal survey about PSI responses and management (via Redcap, web-based software methodology tool for designing clinical, translational and quality improvement databases: https://project-redcap.org/). Development of survey questions was informed by literature across implementation science (78, 79) and safety science (45, 80–89) domains, as well as team expertise. This survey collected participant demographic details and attitudes towards the patient safety response and review process on a 5-point Likert scale.

#### Qualitative data

After the survey was completed, focus groups and individual interviews were conducted via Microsoft Teams or Zoom software or over the phone following a predefined topic schedule (See Supplementary File S3), recorded and transcribed verbatim. Interviews and focus groups were conducted by researchers (PH (professor; male), MB (PhD, Research fellow; female) and YY (PhD, Research fellow; female)) who are experienced interviewers and health service qualitative researchers. Two of the interviewers (MB and YY) had no prior relationship with the interviewees prior to data collection, however, the lead investigator PH had prior professional relationships with several interviewees, but did not coerce participation. Participants were not reimbursed for their time.

### Data analysis

The survey data was analysed descriptively. PSI investigations are referred to from here as *reviews*. Interview and focus group transcripts were imported into NVIVO v.14 software (90) and thematic analysis techniques (91) were used to guide the iterative inductive analysis of the data. This process was conducted by two researchers (MB and YY), with 25% of transcripts independently double coded to ensure consistency, creditability and rigour (92). The process began with both researchers reading transcripts in full to familiarise themselves with the data, followed by open, line-by-line coding to generate initial inductive codes. These codes were then categorised into broad themes on organisational culture (for example, leadership and patient safety team), the investigation process (for example, who was involved and what methodologies were used), and development and implementation of recommendations. An initial thematic framework was developed collaboratively, grounded in participants' language and recurring concepts across interviews and focus groups (91). The coding framework was refined iteratively through weekly team discussions, where coding decisions, emergent patterns, and thematic boundaries were debated and agreed upon. Discrepancies in coding or theme development were resolved through group consensus to ensure reliability and analytic coherence (92). Following this inductive coding process, the refined set of themes were deductively categorised into the Consolidated Framework for Implementation Research (CFIR) (78, 93). This mapping process involved aligning codes and themes with CFIR's five major domains- intervention characteristics, outer setting, inner setting, characteristics of individuals, and the process of implementation, to better understand the factors influencing how PSI review recommendations are enacted (94). The CFIR framework served as a theoretical lens to interpret and structure the qualitative data, allowing for the identification of both barriers and facilitators to implementation at multiple levels of the healthcare system (94). To deepen insights and strengthen validity through methodological triangulation, the qualitative data were then compared and integrated with quantitative survey findings. This involved identifying areas where interviews and focus group data converged with, complemented, or diverged from survey responses (73). This mixed-methods integration provided a more comprehensive understanding of participants' experiences with PSI reviews and highlighted key contextual and systemic factors influencing the development and implementation of recommendations.

### Ethics approval

The study was granted ethics approval by the Northern Sydney Local Health District (NSLHD) Human Research Ethics Committee (an Institutional Review Board: approval number: 2023/ETH02341) in Sydney, Australia.

## Results

### Quantitative survey

In total, 110 participants were recruited to participate in an interview or focus group, and survey. Participants in Australia (*n* = 99) were based in QLD (*n* = 31), VIC (*n* = 31) NSW (*n* = 29), the ACT (*n* = 7), and one other Australian state (unspecified, *n* = 1), or from international organisations (*n* = 11). Most participants completed the demographic (*n* = 100, 91%) and attitudinal components of the survey (*n* = 99, 90%). Not every question in the survey was answered by participants (see Table 1 for more detail). Participants were from a variety of clinical backgrounds and seniority. Of those who completed the survey, participants were typically from nursing or midwifery backgrounds (*n* = 41, 52%), working primarily in quality and safety/governance roles (*n* = 50, 63%), with more than 20 years' experience working in healthcare (*n* = 47, 60%). The majority of participants were female (*n* = 75, 75%), aged 40–59 years (*n* = 75, 75%) and working in a predominantly non-clinical role (*n* = 67, 85%) (Table 1).

**Table 1:** Participant demographic details.

Characteristics	Details	N	%
**State** *^α^*	Queensland	31	28.2%
	Victoria	31	28.2%
	New South Wales	29	26.4%
	Australian Capital Territory	7	6.4%
	Other Australian state	1	0.9%
	International	11	10.0%
**Gender** ^β^	Female	75	75.0%
	Male	25	25.0%
**Age group** ^β^	Less than 40 years old	16	16.0%
	40–49 years	40	40.0%
	50–59 years	35	35.0%
	60 years or older	9	9.0%
Professional group*	Nursing and midwifery	41	51.9%
	Medical	10	12.7%
	Administration	10	12.7%
	Quality and safety/clinical governance	9	11.4%
	Pharmacy	4	5.1%
	Other *(incl. Allied Health, dentistry)*	5	6.3%
Primary work area*	Quality, patient safety, governance	50	63.3%
	Emergency department	4	5.1%
	Mental health/psychiatry	3	3.8%
	Obstetrics/gynaecology	2	2.5%
	Paediatrics	2	2.5%
	Non-surgical medicine	2	2.5%
	Surgical/perioperative	1	1.3%
	Pharmacy	1	1.3%
	Other *(regional facility office, general ward, no specific unit)*	14	17.7%
Clinical role*	Predominantly clinical role	12	15.2%
	Predominantly non-clinical role	67	84.8%
Length of time working in healthcare*	10 or less years	7	8.9%
	11–20 years	25	31.6%
	More than 20 years	47	59.5%
Role in Organisation*	Senior Manager	29	36.7%
	Middle manager	19	24.1%
	Staff member	17	21.5%
	Line manager	5	6.3%
	Executive	5	6.3%
	Team leader/supervisor	4	5.1%
**Involvement in incident management** ^^^	I undertake incident management and patient safety	57	85.1%
	I co-commission and/or quality assure and/or sign off	20	29.9%
	I am a customer of investigations e.g., clinical directors	13	19.4%
	Other (*review, support, monitor investigations*)	3	4.5%
**Years working in incident investigation** ^#^	10 or less years	13	61.9%
	11–20 years	6	28.6%
	More than 20 years	2	9.5%

several demographic questions were only answered by some participants: ^α^*n* = 110, ^β^*n* = 100, *n = 79, ^*n* = 67, some of which provided multiple answers, ^#^*n* = 21.

Participants were surveyed about their attitudes towards PSI reviews. The majority of participants agreed or strongly agreed that *Investigations are a valuable use of resources for improving patient safety* (*n* = 94/99, 95%), that *consider when work processes and systems may have contributed to an incident* (*n* = 87/100, 87%) and *consider human factors and contextual factors that impact human performance* (*n* = 76/100, 76%). Approximately two thirds or less of participants strongly agreed or agreed that *Actions taken in response to investigations prevent similar incidents from reoccurring* (*n* = 67/99, 68%), that *Clinicians are not unfairly blamed for errors that may have occurred* (*n* = 66/99, 67%) and that *Learning from investigations drives continuous improvement in patient safety* (*n* = 61/100, 61%). Just over half of the participants strongly agreed or agreed that that *Recommendations are accepted and supported by the organisation* (*n* = 58/100, 58%) and *are aimed at the appropriate level (e.g., ward, department, hospital) of the healthcare system* (*n* = 56/99, 57%).

Only half of the participants strongly agreed or agreed that: *Investigations consider what went well, as well as what went wrong* (*n* = 54/100, 54%); that *Healthcare organisations should be able to decide not to investigate an incident if no new information is likely to be gained for learning and improvement* (*n* = 52/98, 53%); that *Learning from previous incidents prevents reoccurrence or repeat incidents* (*n* = 53/100, 53%); and that *Investigators have access to the right tools, guidance, and support to conduct high-quality, systems-based investigations* (*n* = 51/100, 51%), and *have the right knowledge and skills to undertake investigations and recommend change* (*n* = 50/99, 51%). Less than half of participants strongly agreed or agreed and that *recommendations from investigations are achievable* (*n* = 45/100, 45%, while 42% neither agreed nor disagreed).

**Figure 1:**
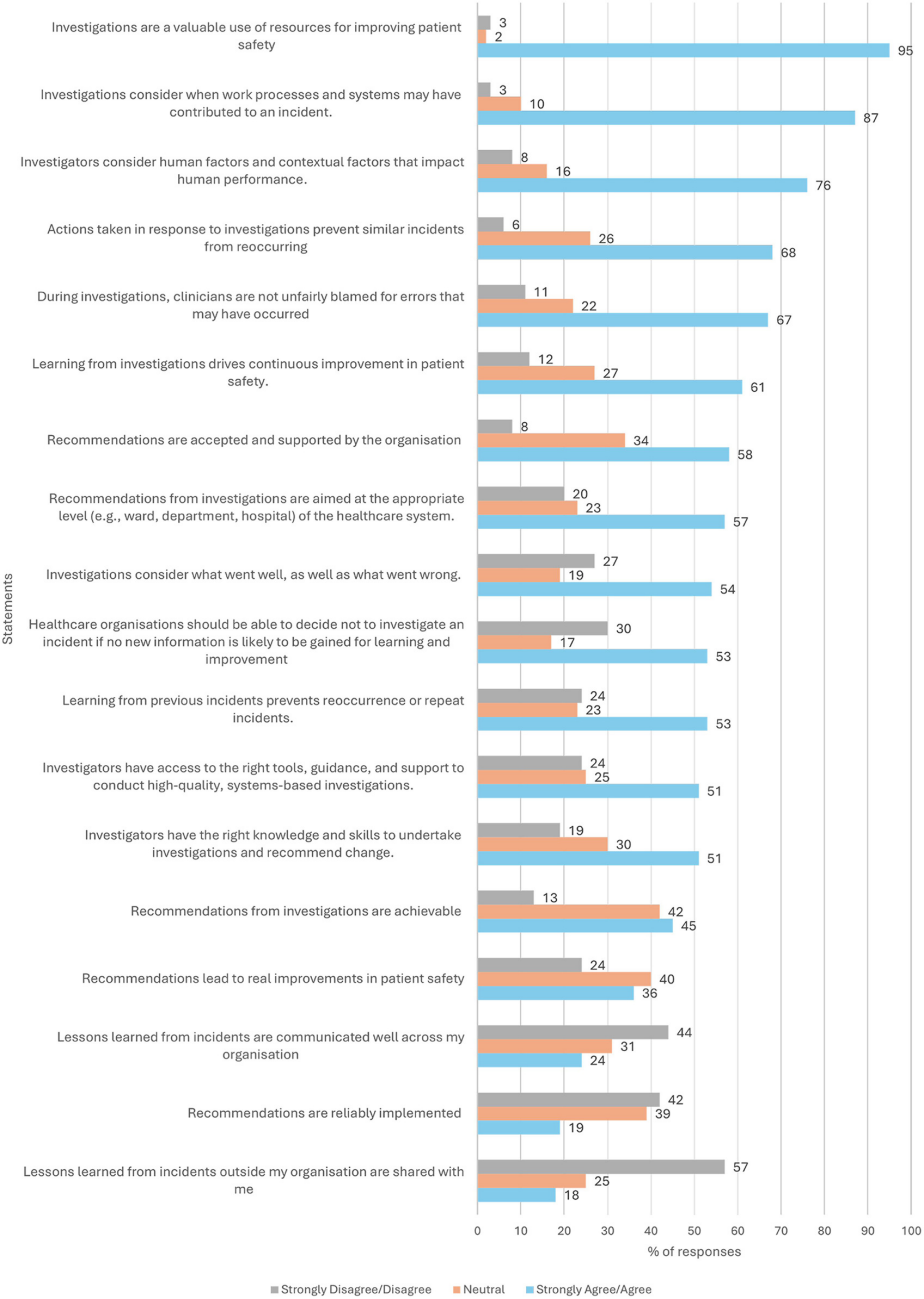
Attitudes towards PSI reviews.

Approximately half of the participants disagreed or strongly disagreed that *Lessons learned from incidents outside their organisation are shared with them* (*n* = 57/100, 57%), that *Lessons learned from incidents were well communicated across their organisations or shared from outside their organisation* (*n* = 44/99, 44%, while 31% neither agreed, nor disagreed), and that *Recommendations are reliably implemented* (*n* = 41/98, 42%, while 39% neither agreed, nor disagreed). Most participants neither agreed, nor disagreed that *Recommendations lead to real improvements in patient safety* (*n* = 40/100, 40%, while 36% agreed/strongly agreed) (Figure 1).

### Qualitative data

**Figure 2:**
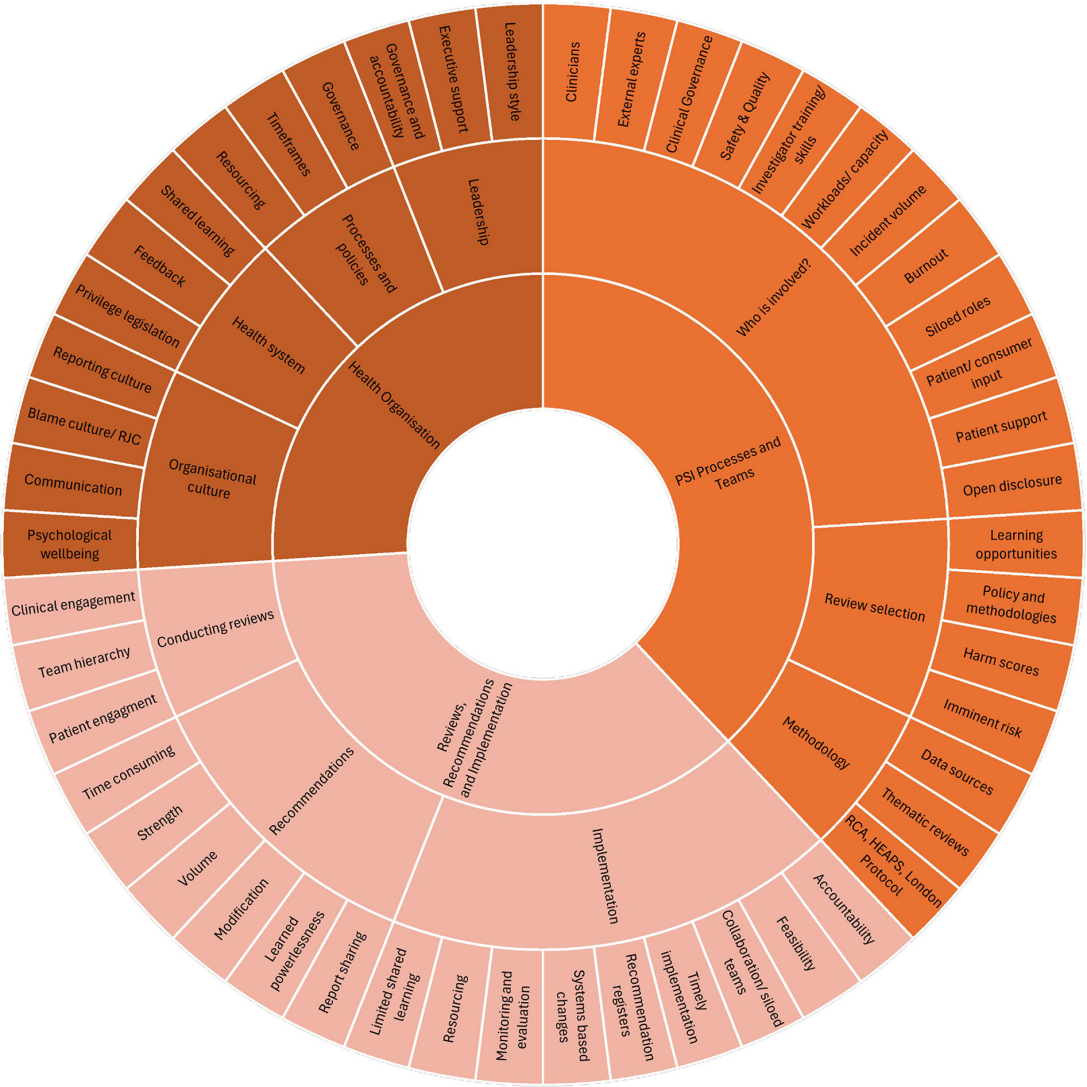
Themes and subthemes from the thematic analysis of the interview and focus group transcripts of the PSI response process.

Individual interviews (*n* = 32 participants) or focus group (*n* = 78 participants across 25 focus groups) were conducted with participants across Australia (*n* = 99) (Victoria (*n* = 31), Queensland (*n* = 31), New South Wales (*n* = 29), the Australian Capital Territory (*n* = 7), and one other Australian state (*n* = 1)), and internationally (*n* = 11) (across the United Kingdom (*n* = 6), New Zealand (*n* = 3), Japan (*n* = 1), and Canada (*n* = 1)). Each interview and focus group ran for approximately 60 min, equalling a total of 57 h of data collection. Results from Australia and the international cohort are presented together. The thematic analysis of the interview and focus group transcripts identified key strengths and challenges of the PSI response process across three key themes: Selection of PSI Reviews; Reviews, Recommendations and Implementation; and Health Organisations and Wider System Influences (Figure 2). Exemplar quotes supporting the themes and subthemes are included. To maintain anonymity, quotes are labelled using participant codes and demographic data have not been presented alongside quotes.

### Selection of patient safety reviews

#### How PSI reviews are selected

Participants indicated that all reported PSIs are assessed in one form or another, depending on the severity and outcome of the incident, to learn from and prevent recurrence. However, the depth in which they are reviewed is variable. Imminent risk is reviewed, and strategies put in place to prevent the incident from reoccurring in the short term. Participants discussed how the PSI review process is typically guided by triage using the incident harm score, as to whether the incident requires in-depth investigation or a more rapid review. The treating clinician or nurse reports the incident, within incident management software, and the severity score is assessed and verified. Participants noted that there was often misunderstanding of the criteria for different harm scores by the reporting clinician, resulting in more incidents being categorised as having a more severe harm score than necessary. Participants also noted potential for conflict of interest when line managers verify harm scores. They explained that incidents, initially rated as severe or serious (e.g., with a *SAC, ISR* or *Harm Score* 1 or 2), are typically triaged by an executive director, reviewed and finalised by an executive committee, and that harm scores are commonly revised to a lower harm level. Participants described how committee meetings may discuss a range of incident types with different severity levels. In addition to validating the harm score, they decide which methodology will be utilised to investigate the incident and assign review team members to conduct the review. It was highlighted that this executive review can be delayed, however, if executive staff are time poor.

Participants detailed how lower severity incidents may be reviewed by a Morbidity and Mortality review team (M & M) meeting, or a local line manager, discussed at Grand Rounds, or reviewed via a desk top review by the quality and safety team or a local ward/department clinical manager. These incidents are typically reviewed through a rapid review methodology or not at all in any meaningful way. If there is significant opportunity to learn from the incident, then an executive team may commission the patient safety team to lead a more in-depth review. Consideration may also be given to potential for harm, particularly in near miss events.

Participants explained how across different jurisdictions, policies dictate whether incidents with high harm scores are mandated to be reviewed using specific in-depth review methodologies [such as a Serious Adverse Event Review (SAER) for harm score 1 incidents in NSW] (17). They noted that this process may not specifically consider whether a comprehensive review would provide opportunities to learn from the incident. When not bound by such legislation or policy, the opportunity for learning from the incident is often considered to select the most appropriate review methodology.

Participants reported that serious incidents including those resulting in death or permanent harm make up a small proportion of incidents that occur annually but consume the vast majority of resources to conduct in-depth reviews (often in response to legislation) that tend to be reactive, case specific and focused on behaviour. They identified that this limits the opportunities and resources to review and learn from lower harm incidents, and near misses through proactive and preventative improvement work that aligns with a systems-thinking approach. For certain incident types, such as falls, or when a system wide issue is identified (similar events occurring across a hospital), participants explained that a rapid or thematic review may be conducted. Compared to an in-depth review, many felt that this rapid review option has the benefit of releasing resources for other in-depth investigations of serious harm events, or lower harm events and near misses, as well as the implementation of review recommendations.

“Someone in their 90s who’s had a fall in the hospital…not saying we don't review those, but can we do that through a one pager checklist, which is kind of what we did in COVID. We very much want to free up for those [reviews] that really have a lot of opportunity for learning” Participant 19, NSW

#### PSI review methodology

Participants discussed how they utilise various investigation methods during PSI reviews. Commonly described methodologies included Root Cause Analysis (RCA), the London Protocol, Human Error and Patient Safety framework (HEAPS), Root Cause Analysis and Action (RCA2), Concise and comprehensive reviews, and the Accident Mapping Approach (AcciMap), as well as methods such as audit and feedback, cause and effect analysis (Fish bone diagrams or Ishikawa analysis), Five Whys analysis, and more rapid processes of review by Morbidity and Mortality teams using a template. In our study, RCA and RCA2 remain the dominant method in many Australian states and territories. The international participants also reported using Functional Resonance Analysis Method (FRAM), Systems Theoretic Accident Modelling and Processes (STAMP), Hierarchical Task Analysis (HTA), Systems Engineering Initiative for Patient Safety (SEIPS), The Patient Safety Incident Response Framework (PSIRF), as well as the Yorkshire contributory factors framework and the Learning Review Methodology (See Box 2).

Box 2 **Overview of Common PSI Review Methodologies**

**Table:** 

Methodology(68)	Full Name	Primary focus	Theoretical underpinning	Further information
RCA	Root Cause Analysis	Identifies causal factors using linear, event-based reasoning.	Accident causation models, “human errors” are rooted in defective systems and controls. Systemic analysis with structured recommendations(45)	NSW Clinical Excellence Commission (CEC) (142)
RCA2	Root Cause Analysis and Action	Enhances RCA with a stronger focus on actionable, systems-level improvements.	Accident causation models, “human errors” are rooted in defective systems and controls. Systemic analysis with structured recommendations and theory-based change implementation. (143)	AHRQ (USA): *RCA2: Improving Root Cause Analyses and Actions to Prevent Harm*. (144)
London Protocol	–	Systems-based, compassionate, inclusive approach, including stronger patient and family engagement, support for all affected parties	Reason's Swiss Cheese Model; systems approach. (58)	Taylor-Adams & Vincent (2004). (25) Vincent et al., (2025) (58)
HEAPS	Human Error and Patient Safety Framework	Categorises contributing human and system factors.	Human factors, Reason's model. (145)	Queensland Health, Australia. (146)
SEIPS	Systems Engineering Initiative for Patient Safety	Analyses the interaction of system components: people, tools, tasks, environment.	Human factors; sociotechnical systems theory.	Carayon, P., et al. (2006) (147), Holden, R.J. et al. (2013) (85), Carayon, P., et al. (2020) (148), Holden, R.J. et al. (2021) (28)
STAMP	Systems Theoretic Accident Model and Processes	Focuses on control structures and constraints within complex systems.	Systems theory; safety control models.	Leveson, N. (2016). (149)
FRAM	Functional Resonance Analysis Method	Examines variability in daily work that may contribute to outcomes.	Resilience engineering; complexity science.	Hollnagel, E. (2017). (30)
HFACS-Healthcare	Human Factors Analysis and Classification System (adapted for healthcare)	Classifies latent and active failures across organisational levels.	Human error taxonomy; aviation safety.	Diller T. et al. (2014) (33), Paterson, E., et al. (2024) (150)
AcciMap	Accident Mapping Approach	Visual mapping of systemic failures across organisational levels.	Rasmussen's risk management framework. (151)	Branford et al. (2009). (152)
FMEA	Failure Modes and Effects Analysis	Proactively identifies and ranks potential failure points in a process.	Engineering risk assessment.	Davis, S., et al. (2008). (153)
SAER	Serious Adverse Event Review	Used in Ireland and Scotland; blends RCA with wider systems focus.	Root cause plus systems-level thinking.	HSE Ireland, NHS Scotland. (154)
Concise Review	–	Used for no/low harm events to allow rapid reflection and learning.	Abbreviated investigation model.	NHS England (as part of PSIRF guidance). (155)
Comprehensive Review	–	Used for more serious events with wider stakeholder input.	Structured system review.	NHS England (PSIRF). (155)
Aggregate Review	–	Reviews multiple similar events for systemic themes.	Trend/systemic analysis.	PSIRF and some state-based systems (e.g., NSW). (156)
PSIRF	Patient Safety Incident Response Framework	Risk-proportionate approach to investigations; supports learning, not blame.	Principles of safety science, restorative justice.	NHS England, 2022. (18)

Sources: as shown; Authors’ conceptualisation.

There was no consistent methodology reported to work well across different incidents. RCAs were perceived by participants to have limited effectiveness, learning opportunities and capacity to share findings, and incidents reviewed by RCA (typically high severity) were often protected by legal privilege legislation. Thematic reviews of high frequency and similar events that occur across a hospital were perceived to achieve change in practice and patient outcomes and seen as helpful tools to analyse commonly reoccurring incidents from a systems perspective.

“So, you know because SAC 1 is a myopic view of an incident that’s occurred to typically one person or a family…If they are aggregated then it tends to help change systems” Participant 30, QLD

A small number of participants described conducting thematic reviews themselves, with some noting that if a patient's family wanted individual findings and action, then an individual comprehensive review was typically conducted. Participants however felt that individual reviews often did not effectively capture contextual data around the incident. They noted that reviews in Australia tend not to undertake secondary observations of standard clinical practice and tend to rely on interviews and medical records as their key data sources, whereas in the UK, observations have become a well-regarded source of data to inform incident reviews.

“The observational work offers a lot more to us because I think that’s our segue between being a very traditional, ‘What happened to this person and why?’, to a broader understanding of ‘How do things normally work and how would you factor that into this thing happening?’’ Because that thing wasn't unique. That could have happened any day. It just seemed to happen this once and it could happen again. Why? So, the observation work, makes a difference in whatever we do. It’s really important” Participant 31, International expert (Healthcare leader, United Kingdom)

#### Who is involved in patient safety incident reviews?

Participants described how PSI review teams may be commissioned by a senior executive such as the Chief Executive Officer, or an executive triage team. Establishing an investigation team was seen as a fundamental step in the PSI review process as it requires careful planning for balance of clinical specialty subject matter expertise, experience and independence, and is time consuming. Getting buy-in from busy, senior clinicians, nurses and heads of units was identified as challenging with operational demands given precedence. Participants described how the ideal PSI team would include independent experts external to the organisation, but that recruitment is reportedly variable and challenging for those roles. Participants felt that independent panel members can bring expertise around systems thinking and human factors, dedicated focus, and encourage accountability and professionalism.

“[External experts] bring some expertise around systems thinking, around human factors. It also brings in a level of accountability. Everybody kind of behaves in the room well and follows the process a little bit better if there’s somebody external that they think is watching them” Participant 22, VIC

Participants also highlighted that proxy representation of the individual staff involved in the event (staff in similar roles with similar levels of experience) may also be included in the review team. While engaging with and interviewing individual staff directly involved in the incident provides valuable insight into what occurred, concern was noted that their perspective may bias a review, by reducing objectivity, and thus their inclusion on the review panel should be avoided. Participants felt that the chair of the PSI review panel should have human factors knowledge and systems-thinking experience, as well as clinical expertise relevant to the incident, without having been directly involved, as they play an important role to facilitate input from panel members in a non-hierarchical manner. Support by administration staff was also perceived to be beneficial for reviews teams, to reduce the logistical and organisational burden involved with conducting an incident review.

To be a skilled investigator, participants felt that individuals need clinical knowledge, expertise in systems thinking and human factors as well as a strong understanding of investigation methodologies and general principles. Review team members, however, were often perceived to lack experience and knowledge of those paradigms. Training and experience of review team members was reportedly variable across jurisdictions, partly influenced by high staff turnover, and limited training resources and courses available. Participants noted that inexperienced team members need additional training and support to conduct reviews and produce high quality reports with Specific, Measurable, Achievable, Relevant and Time-bound (SMART) recommendations. They described the same challenges for inexperienced quality and safety staff who require on the job training, adding to the workload of quality and safety teams. Staff working in higher volume metropolitan hospitals were perceived to have greater incident review experience, compared to lower volume rural and remote sites. More broadly, participants stated that review team members need capacity to conduct reviews, but that this is impacted by high workloads and limited time. Participants also noted staff burnout and frustration that despite effort and emotional investment during the review process, changes to care often do not eventuate.

Safety and Quality and Clinical Governance staff were perceived to provide expert guidance, facilitate reviews for serious harm incidents, and provide support and resources for lower harm incidents. They are also perceived to support the review team once the report is drafted, enabling refinement of recommendations, and providing insight regarding similar concurrent improvements projects to avoid duplication of work and share findings. While safety and quality staff are typically skilled clinically, participants expressed concern that they are not always remunerated in parallel to more high-paying clinical roles, and this in combination with the workload and emotional impact of the job, may contribute to high staff turnover. Participants noted that this high staff turnover in turn translates to constant training of new staff. Safety and Quality and Clinical Governance roles were perceived to have become siloed in some jurisdictions, limiting opportunity to learn from and collaborate with each other*.* Participants noted that a strong community of practice is needed, but not always available, to support and facilitate collaboration across (siloed) quality and safety teams.

Depending on the jurisdiction, participants reported that some review panels include consumer representatives (CRs), particularly in Victoria where it has been encouraged in policy. Participants generally considered CRs to be a valuable addition to the team, bringing a different perspective to the review. However, participants noted that CR involvement requires careful facilitation, executive support and training both for the CR, and the review panel. Participants described how effective engagement with CRs requires facilitation by the review panel Chair to ensure the wellbeing and psychological safety of the CR and the panel staff. Consumer input was considered useful by participants, even if the consumer is not directly on the review panel, with consumer review of reports and findings to be helpful.

Engagement with, and seeking input and feedback from, patients and family members about the PSI and review process was reportedly variable, but valued, adding depth to the review if facilitated sensitively and supported through staff training. Participants described how this may be conducted by a dedicated family contact (DFC, in New South Wales for serious incidents), a liaison staff member dedicated to support families, or a social worker, who aim to engage with patients and families, support them throughout the review process, and act as a point of contact. Participants noted that DFCs should also provide additional information around the review progress, findings and actions, as well as linking families to additional support services. However, this is variable across jurisdictions and was identified as an area for improvement.

*Clinician disclosure* to patients and families is conducted by the treating clinician, typically shortly after the incident. Participants noted how important this step is, but how variable the quality of the disclosure can be, influenced by the level of staff experience, training and confidence, as well as the organisational culture around clinician disclosure being conducted after an incident. Participants described how o*pen disclosure*, a process which includes an apology, is then conducted by independent staff members, such as quality and safety or executive clinical directors, typically before the review process begins. This process is embedded in the mandated Victorian Statutory Duty of Candour process, which participants noted has improved engagement with consumers. It was noted that patients and families may then be provided with a second open disclosure process about the review and findings, once complete.

### Reviews, recommendations and implementation

#### Conducting reviews

The key purpose of a PSI review is to identify what went wrong, to learn from the incident and to prevent reoccurrences by identifying and implementing recommendations. Consulting with staff who were involved in the incident, as well as those likely to be affected by the recommendations including medical directors with oversight of staff and teams, was deemed useful, but variable in practice. Clinical engagement during the PSI review process was identified as a key challenge, when medical staff are reluctant to participate in the review process. Participants posited that this may be related to the Visiting Medical Officer (VMO) model, particularly in rural and regional areas, where clinicians are appointed to teams for short periods of time and therefore are not well integrated into the local system.

Consulting with the teams directly involved in the incident, and those who will be impacted by the recommendations was considered important, to capture their perspectives, and to support them after the incident, particularly when staff distress is identified. Participants felt that engagement with staff directly involved in the incident [acknowledging the *second victim* experience (95, 96)], needs to be managed carefully however, as the process of review can add to their trauma. Consulting with safety and quality or consumer engagement staff who are not directly involved in the review, was also identified as a useful quality check process to ensure rigour in the review process and findings.

Team hierarchy was perceived to influence the recognition of some review team members, influencing their input into report findings. Another challenge noted by participants was the introduction of new incident management or Electronic Medical Record (EMR) systems. According to participants, if staff do not know how to navigate new systems well, information may not be captured adequately, impacting the quality of analysis in the review.

In terms of engagement with patients and families while conducting the review and developing recommendations, participants reported variation in the formal processes used to hear the patient perspective, and what they want the review to achieve, as well as how this is considered and documented within the review report. This was identified as an area for improvement by most participants. Participants raised concerns about patient and family vulnerability and how this perception can lead to their exclusion from participation in reviews, though this remains a subject of ongoing discussion.

#### Developing recommendations

Participants posited that effective recommendations reflect a thoughtful and in-depth analysis of the contributing factors, address system and process change and clearly link to appropriate implementation strategies. Developing meaningful, concise, clear, and effective recommendations that are measurable, achievable and implementable, was identified as a challenging, time consuming, resource intensive process, requiring skill and engagement with large clinical teams**.** Participants felt that following SMART (Specific, Measurable, Achievable, Realistic and Timely) principles can support the development of quality recommendations. Consulting with past reviews of incidents (both locally, intra-state and nationally) was also considered to be helpful in learning about effective recommendations. However, this can be challenging as it relies on individual networking, with no current infrastructure available, or secure permission system, to search for such information easily.

Participants described how clinician workloads and lack of time to conduct the review and formulate recommendations can limit the quality of outputs. Participants also noted it was challenging to get busy clinicians to engage in review training to further develop skills. Development of recommendations by team members without relevant clinical experience or without consultation with relevant clinical staff was perceived to impact the applicability of recommendations. This was noted as a particular issue in rural settings, when external but non-rural clinicians may develop recommendations without contextual understanding of the setting, limiting the applicability of their recommendations.

Participants noted a tendency to develop weak and ineffective recommendations and attributed this to the fact that recommendations are an expected review component, and team members feel compelled to make recommendations. They felt that the resulting large volume of weak, standard, “go to” recommendations (such as to change a policy or educate a staff member or specified group) have resource implications by detracting resources from the implementation of higher quality recommendations. Participants described how clinicians may lack implementation science training/expertise limiting their use of evidence-based recommendations and behaviour change techniques. Participants noted that there are efforts in some jurisdictions to minimise the number of recommendations and ensure final recommendations are concise and strong.

Participants explained how incident analysis reports and recommendations are typically reviewed at various executive levels, modified as appropriate, finalised, endorsed by a general manager or clinical director, and signed off by a senior executive. Support and engagement with quality and safety teams, executives and leadership prior to formal sign off meetings was seen to support quality recommendation development. This process was also seen to help refine and strengthen recommendations, to ensure they are effective and sustainable, provide expert but independent advice and alert teams to similar recommendations or improvement work implemented elsewhere within the organisation, and to minimise the timeframe for signoff by executive.

On the other hand, participants noted that recommendations may be amended and weakened at the executive endorsement and sign off stage. This ‘sense of learned powerlessness’ was a common challenge discussed [a term that originates from the psychological phenomenon of learned helplessness (97)], when an issue and solution is identified, but no corresponding recommendation is made in the final report because system and resource-intensive recommendations are often seen as unfeasible, unrealistic, or unlikely to be approved by executives. Participants felt that they had limited authority to recommend meaningful changes. Participants reported that PSI review reports and findings are often influenced and modified by executives who are conscious of political or media pressure, as well as concern about reputational damage, or that certain recommendations could be interpreted as criticising health service organisations. They felt that such reactive responses to political and social influences can influence review processes, result in weaker recommendations and undermine the aim of the process. For example:

“You then go back with the draft report to the executive and more often than not, the initial response is defensiveness. ‘No, you can't say that. No, did you really find that? And I don't think you've shown that, have you’, regardless of the methodology that was used and the evidence that’s presented. I think that it’s human nature, right? People don't want to hear that there’s wrong in their house. And especially if the answer is that there’s going to need to be an investment in resources into trying to fix it. It’s very difficult to create change. And I think that boards and executive don't necessarily recognise that that change has to come from the top. It’s always a sort of a look down focus. ‘OK, how do we make these people at the at the pointy end work harder and work better?’” Participant 9, VIC

There was large variation noted as to whether review reports (or their drafts) are provided to patients and families, or findings simply summarised, with participants reporting that some organisations only provide reports to families following a Freedom of Information request, while others share both the draft and final reports. Participants noted that reports about higher severity incidents were often shared with families, however, reports or summaries of findings for lower severity incidents are typically not shared. Participants felt that sharing the draft report with families provides an opportunity to gather their perspective and input on the findings and recommendations, and the inclusion of a plain language summary can be beneficial. Several participants noted that often reports are not well received by families, as they inadequately capture the family's experience and trauma. Consideration of the benefit or impact of an incident review for patients and families, as well as staff wellbeing, was also considered important.

#### Implementing recommendations

Participants described how report findings and recommendations are uploaded into an incident management system and a timeframe is set for implementation based on the KPIs and policy of each jurisdiction. The implementation process may then be tracked and monitored through that system by safety and quality teams who are typically not tasked with responsibility for implementation, however participant responses suggest this is variable. Participants explained that in some jurisdictions, quality coordinators coordinate with clinical business units to facilitate the implementation of recommendations, document and monitor the process, and provide evidence back to the units. They noted that recommendation implementation may be monitored by executive committees and safety and quality teams, reporting on the progress and completion as appropriate. Participants reported this process to be variable in Australia and internationally with poor monitoring negatively impacting on effective implementation, sustainment and achievement of change. They posited that this may be as the development of recommendations and their implementation may be tasked to different siloed teams, limiting follow through and accountability. Participants were enthusiastic about the benefits of recommendation registers to support the tracking and monitoring of the implementation of recommendations, particularly when accountability was tasked to a position, rather than an individual, to account for staff turnover. Assigning recommendations and their implementation to appropriate senior staff who are responsible for the achievement of change, and who can delegate the process, was considered an important facilitator to this process. However, this was also noted as challenging when recommendations relate to multiple areas in the health service as opposed to a single ward.

When implementation of a recommendation is monitored, participants highlighted that evaluation to ensure fidelity (implementation as intended) and assessment of the quality of outcomes is variable**.** Quality monitoring and evaluation were considered important to establish evidence regarding strategies that successfully impact care and patient outcomes to garner support for sustainability and scale up of the change, or to apply improvement strategies to other areas. Dashboards tracking recommendation implementation, regular meetings, and biannual reports were perceived as ensuring accountability and support implementation:

“My level of confidence is growing. SAC 1 and SAC 2 recommendations are monitored in a live dashboard that is able to be searched by each of our directorates… We monitor it through the Executive Safety and Quality Committee, and they're also internally monitored by the Directorate Safety and Quality meeting…Whilst I have reporting that says someone has signed off that the recommendations have been implemented, we're not great at having a checking process to check that that’s true. And we had an internal audit process in the last 12 months that showed us that we were not, and that we need to actually implement that process of an audit process more effectively. We're working on what that might look like at the moment. But I don't believe, given that many of the old recommendations were nonsense, I don't believe most of them had been properly implemented at all” Participant 36, QLD

According to participants, the implementation of recommendations associated with serious incidents tends to be tracked; however, this is often not the case for lower harm incidents. Participants noted the high frequency of particular incidents reoccurring, resulting in similar review recommendations being developed but not effecting change, and how this indicates a need to enhance the quality of recommendations and their implementation. Implementation was perceived to have fewer resources and time allocated, in comparison to the review process. Participants noted that this results in recommendations and their implementation not achieving intended changes to care practices and patient outcomes. Similarly, participants reported that it can be challenging when funding dedicated to implementing a specific recommendation ends.

Participants explained that nurses in leadership roles (for example, Clinical Nurse Consultants and Nurse Unit Managers) or clinicians, are often tasked to implement recommendations in their teams, or areas, despite not always being familiar with the context, incident or contributory factors underpinning the recommendation. Hierarchies and power dynamics across teams were perceived to make it challenging to establish clinician buy-in with the implementation of recommendations, with both issues challenging engagement and the potential to influence change across a hospital. Greater collaboration and interdivisional agreement on implementation action plans was perceived to improve buy-in and holistic understanding of the drivers of change. Participants reported that high staff turnover also leads to loss of institutional knowledge and understanding of the review underpinning the recommendations, impacting the implementation of recommendations. While recommendations and implementation plans may be documented within the original reporting software, long-term monitoring of the implementation of the recommendation, and outcomes achieved was reported to be variable, particularly after the implementation process has been signed off.

Frustration was reported that recommendations and their implementation strategies get modified through various bureaucratic processes. Final implementation plans were often perceived to differ to what was intended by the review team, limiting the accountability for effective implementation. Staff turnover at the management or executive level was seen to contribute to this, leading to limited accountability when implementation is person specific, rather than role specific. Participants felt that management and executive role-accountability supported implementation and facilitated change. Participants also highlighted a tension between being reactive to an incident while also providing enough time for a quality review and implementation process. Timely but effective implementation of recommendations was considered fundamental, to ensure those involved feel confident they can enact change.

Weak recommendations that are not specific or measurable, were seen to be challenging to follow up and implement, making implementation an ineffective tick box exercise that rarely achieves the intended change to care practice or patient outcomes. However, participants reported that even strong recommendations may be poorly implemented with limited support and expertise to guide the process. Despite the weak nature and limited evidence for education alone as a behaviour change strategy, participants described how education-based recommendations are often recommended. This is in favour of more challenging systems thinking-based recommendations. Participants felt that system-wide changes are particularly difficult to implement, as they involve cooperation and coordination of multiple interdivisional and inter-organisational stakeholders, particularly in jurisdictions with devolved health services. They were also perceived to be more resource intensive, and therefore not always perceived as feasible. Participants noted limited shared learning about recommendations and implementation strategies that have been effective elsewhere. Participants noted that access to this information would be useful to support the development of more robust implementation plans. Providing patients and families with updates on the progress of implementation, sometimes as a part of the open disclosure process, was also variable, as staff felt some families were not ready to engage in the process, whereas others were keen to be updated.

### Health organisation

#### Organisational processes and policies

Participants reported that there were generally clear, mandated review, governance and accountability processes to follow for incidents. They held that regular quality and safety meetings provide support to collaborate, and monitor, conduct preliminary assessments, assess the review criteria thresholds to determine the required methodologies for incident reviews, and support open disclosure and implementation of recommendations processes.

A commonly reported challenge for participants was the strict timeframes and associated Key Performance Indicators for the PSI review process to be initiated and completed within. Participants noted in QLD, for example, reviews of PSIs with a Severity Assessment Code 1 (SAC1) [equivalent to Harm score 1 in NSW, or Incident Severity Rating (ISR) in Vic] must be completed within 90 days. These timeframes were described as being unfeasible for overworked staff, particularly for complex incidents involving multiple clinical specialties requiring enormous coordination and engaging with an often part-time workforce. Resourcing challenges were discussed regarding a lack of staff and time, challenging and delaying the completion of reviews and findings. On the other hand, deadlines were also perceived to keep participants accountable. Adding to these challenges, the reporting management systems commonly used were not considered to be user friendly.

“Sometimes they're really rushed at the end of a process because the 60 days is so tight. So, there’s a risk there. And, and there’s been conversations definitely at the DCG level [Director of Clinical Governance—a role at a health service] around wouldn't it be wonderful if we had 60 days to do the findings report and then another month to do the Recs [recommendations] so we could really put some nice thought and planning into the recommendations. Often, they are weak if we look at sort of the hierarchy of recommendations and then they're often not implemented or the process for follow up or sustainability is weak” Participant 11, NSW

The value of undertaking individual reviews for certain incident types (such as falls) was queried, with participants expressing frustration at the resource intensive comprehensive review process that often results in limited learning. Participants explained that policies to assess the need for a comprehensive review lacked flexibility, leading to duplication of work through individual reviews of similar events, without learning from previous reviews, suggesting in some circumstances cluster/thematic reviews may provide greater value.

#### Broader health system

Similar to local organisations, participants reported limited sharing of learnings across health services, with siloed services and clinicians unaware of incident reviews and implementation strategies conducted elsewhere, and limited opportunities to learn from exemplar reports from inter and intra-state. Participants noted that certain jurisdictions are also bound by Qualified Privilege or Statutory Immunity legislation, which limits the capacity to share findings. Some participants reported limited comparative feedback from statewide or national departments of health about the quality of the review report and findings, limiting staff opportunities for learning,

“We don't actually get feedback from [statewide bodies] to say ‘We really liked what you did here’… I think that’s one of the failures in the systems of why it doesn't get better because we're just not getting that feedback or shared learning” Participant 14, (state suppressed)

#### Leadership

Participants indicated that leadership style influences the incident review process and culture around reporting. Transparent and collaborative leaders, with dual governance or safety based leadership and clinical roles support the culture of reporting PSIs, as well as systems thinking, while minimising blame and focusing on opportunities to learn from incidents and near misses. Executive support was considered important, providing broad oversight of the incident review process and guidance through legislative requirements, supporting communication with families, and endorsing review reports. However, participants also felt that executives were cautious of political influences, and wary of identifying contributory factors to an incident in a review that could be interpreted as criticisms of health departments. Similarly, participants identified that executives may amend final recommendations that have an operational impact and require resourcing for improvements, resulting in weaker recommendations.

“There’s often a fair amount of management input to reshape the recommendations. [They] tend to get a bit … modified by the time that it actually gets to the [Statewide body] … anything that involves resourcing. So, the overarching challenge with all of this is that the people who are conducting the reviews are constrained by the fact that many of the recommendations that they would like to make have operational impact and resourcing impact” Participant 2, VIC

A robust governance and accountability system was seen as a key strength to support the development, implementation and evaluation of evidence-based recommendations that are likely to be effective and sustainable. International experts identified this as an area to focus improvement efforts on, to enhance reporting, analysis and accountability, and reduce the siloed nature of services.

“It’s quite siloed. There’s so many statutory bodies set up at different levels of a system, but all statutory bodies and the responsibility and accountability and escalation and management of risk is very much more muddied” Participant 3, International expert (Policy advisor, New Zealand)

#### Organisational culture

An open, transparent and restorative just culture with a systems thinking focus was touted as a fundamental element to patient safety, to encourage reporting with timely feedback. A positive reporting culture was perceived to be improving across Australia and internationally, albeit slowly, encouraging staff to be open about incidents, but needs continuing investment to develop further. Participants noted that improvements to this culture can be hindered by staff turnover. Resistance to incident reporting remains a challenge, with participants noting under reporting or insufficient escalation of incidents, partly a result of staff not understanding the escalation and governance processes.

Participants highlighted the importance of listening to consumer voices was as component of a just and learning-oriented reporting culture. From their perspective, meaningful involvement of consumers and their families in the review processes should be carefully supported and embedded, not as a procedural task, but as an ethical commitment. Several participants acknowledged that complexities involved, including the vulnerability of those affected. Similarly, ensuring that clinician disclosure is conducted consistently, and that clinicians are well trained and consistently supported to conduct disclosure. was identified as another important improvement that needs to be embedded within the review process. Some participants noted that while strengthening a strong culture of reporting is important, if not managed carefully, it may also increase the workload of quality, safety and improvement teams.

“We’ve really got good clinician engagement in open and transparent reporting. So, we feel like we are almost having a bit of an over reporting process at the moment. People are putting in absolutely everything and we're having to really review and peel back” Participant 5, VIC

Participants discussed how many health services still face the challenge of overcoming a blame culture, embedded from the executive level down, and strive to ensure that the restorative justice message is consistent at all levels of the organisation. Poor and inconsistent communication, a lack of transparency and prevailing blame language were perceived to counteract efforts to bring about organisational cultural change, and efforts are required to address misconceptions staff hold about punitive consequences of reporting incidents. This is particularly challenging when staff are overworked.

“One in four or five people on the panel will be someone who we need as a subject matter expert, but will be coming with that perspective of, ‘This is just because Susie made that mistake’” Participant 4, VIC

Participants felt that some individuals and organisations harbor concerns about reputational damage resulting from PSI reviews. A lack of psychological wellbeing support for staff conducting the reviews or involved in the incident was also noted. In some cases, the trauma of the incident, and the review process, was reported to result in staff leaving their positions, losing valuable human and organisational knowledge in the process.

“There’s a reluctance and a fear of reputational damage in healthcare, and people don't want to be seen to be getting things wrong. And then that’s going to stifle the learning. And I think that’s just a cultural thing, which is a very big challenge to overcome” Participant 3, International expert (Policy advisor, New Zealand)

This lingering blame culture was seen to result in limited opportunities for shared learning from incidents, and near misses. Participants noted that learning can be further limited by the siloed nature of health services and teams leading to duplication of improvement work, with poor dissemination of findings from reports or thematic summaries of incidents, and limited sharing across teams, organisations, and health systems. Participants also felt that sharing can also be hindered by confusion around Qualified Privilege or Statutory Immunity legislation (98) and the limitations that it places on data sharing. Participants noted that findings from reviews are often not shared with the staff who reported the incident, which may discourage staff from reporting future incidents if they feel nothing was actioned by their report. Similarly, quality and safety teams may share findings from reviews, or thematic analyses with executive or senior staff, however participants noted that this information may not reach the clinicians on the ground who may be impacted by the implemented recommendations, nor the reporting clinician or clinicians who were directly involved in the original incident.

### Comparison of qualitative and quantitative data

Survey data and interview and focus group data were compared and triangulated to provide a more in depth understanding of the key concepts. These findings were generally concordant or complementary (describing different facets of themes) (73). There were a few discordant findings: In the survey, most participants agreed that *Investigations consider when work processes and systems may have contributed to an incident* and the *Investigators consider human factors and contextual factors that impact human performance*. These were two points that participants highlighted as needing further development in the interviews, suggesting that systems thinking and human factors, and broader system change were not always adequately considered or reflected in PSI review recommendations. Similarly, the interviews identified that many reviewers felt despondent that implementation of recommendations resulting from PSI reviews was often ineffective, whereas two thirds of survey responses agreed that *Actions taken in response to investigations prevent similar incidents from reoccurring*. Two thirds of survey responses felt that individuals were not unfairly blamed during the review process; this may still be an area needing further development, with interview findings highlighting an ongoing culture of blame in some organisations. These discordant findings may reflect both how participants view the purpose of PSI reviews identified in survey responses, as well as the practical challenges to achieving those purposes identified in the interviews and focus groups. It is also worth noting that findings from the Australian cohort, across the four states and territories were largely aligned with each other and with responses from the international cohort.

The key themes and subthemes have been mapped against the CFIR framework (93) to systematically analyse the key strengths and challenges to PSI reviews and implementation of recommendations. This process categorises and highlights key elements that are likely to influence the effectiveness of incident reviews across five domains: Characteristics of patient safety review process and implementation of recommendations (Innovation domain), External environment and context (Outer setting domain), Internal context (Inner setting domain), Characteristics and attitudes of practice staff and clinicians (Individual domain), Patient safety review implementation (Implementation process domain) (Table 2). These key determinants will inform the proceeding study to codesign a system-wide improvement to support the PSI review process.

**Table 2:** Application of CFIR components to highlight facilitators and barriers to implementation of patient safety investigations [based on model by Keith 2017 (93)].

Domains	Themes, Subthemes and codes	
	Strengths	Challenges
Innovation domain	Selection of PSI Reviews - How reviews are selected • Thematic reviews enable resources to be used for in depth investigation and implementation of recommendations related to serious harm events, or to investigate near misses more consistently Selection of PSI Reviews - Data analysis methodology • Thematic analysis of incidents are helpful tools to analyse commonly reoccurring incidents from a systems perspective Reviews in Australia tend not to utilise observations of clinical practice, however they are more common in the UK and provide insight into care processes working well	Selection of PSI Reviews - Data analysis methodology • Participants perceived RCAs to have limited effectiveness, and to be out of date for learning opportunities and sharing of findings, as incidents reviewed by RCA (typically high severity) were often protected by legal Privilege legislation • Reviews often do not effectively capture contextual data about what was happening in the department, around the incident
Outer setting domain	Health organisations and Wider System Influences - Leadership • Robust governance and accountability systems Health organisations and Wider System Influences - Organisational culture • An open, transparent and restorative just culture with a systems focus • A positive reporting culture Health organisations and Wider System Influences - Organisational structural processes • Clear, mandated review, governance and accountability processes to follow for incidents Health organisations and Wider System Influences - Safety and Quality and Clinical Governance staff • A strong community of practice	Health organisations and Wider System Influences - Organisational culture • A strong culture of reporting is important, but can increase the number of incidents reported, impacting capacity to implement changes • Resistance to incident reporting • Blame culture embedded at the executive level • Poor and inconsistent communication, a lack of transparency and prevailing blame language Health organisations and Wider System Influences - Organisational structural processes • Strict review timeframes Health organisations and Wider System Influences - Broader health system • Current legislation frameworks • Limited sharing learning across health service • Limited feedback from statewide bodies Selection of PSI Reviews - How reviews are selected • Policies dictate whether incidents with high SAC ratings or harm scores are mandated to be reviewed using specific comprehensive review methodologies
Inner setting domain (continued)	Health organisations and Wider System Influences - Leadership • Leadership style (Transparent and collaborative leaders better support the culture of reporting) • Executive support Health organisations and Wider System Influences - Organisational culture • Listening to consumer voices Health organisations and Wider System Influences - Safety and Quality staff, clin gov • Safety and Quality and Clinical Governance staff play a key role in guiding and facilitating incident reviews Selection of PSI Reviews - People involved • Open disclosure, is conducted by the treating clinician, typically before the review process begins	Health organisations and Wider System Influences - Leadership • Political influences on executive support Health organisations and Wider System Influences - Organisational culture • Lack of psychological wellbeing support for staff conducting the reviews or involved in the incident • Blame culture and siloed nature of health services results in limited opportunities for learning from incident • Poor dissemination of findings from reports Health organisations and Wider System Influences - Safety and Quality staff, clin gov • High workloads and staff burnout in safety and quality staff • High staff turnover in quality and safety roles, small teams, and the need to train inexperienced new staff on the job • Burnout was enhanced by frustration that changes to care or outcomes often do not eventuate • Improvement efforts were seen to have a reactive focus in response to incidents Selection of PSI Reviews - How reviews are selected • Conflict of interest when line managers are involved in the assessment of harm scores/review processes • Time-poor executive staff delay executive review of the incident and harm score • Privilege legislation impacts the type of review methodology and capacity to share findings Recommendations and implementation - Implementing recommendations • Limited engagement may be related to the Visiting Medical Officer (VMO) model where clinicians are appointed to teams for short periods of time, and therefore aren't as integrated into the local system • Siloed review teams (including clinical governance) who develop the recommendations being separate to the teams who are tasked with implementation of the recommendation • Fewer resources and time are attributed to the implementation process, in comparison to conducting the review process results in recommendations and their implementation not achieving the intended changes, and therefore limited change to care practices and patient outcomes
Individual domain	Selection of PSI Reviews - People involved limited change to care practices and patient outcomes • Senior clinicians, senior nurses included nurse unit managers, and heads of units are often involved in reviews • Independent members bring expertise around systems thinking and human factors and may encourage accountability. • The chair of the review team, who ideally has clinical expertise, but is not related to the space where the incident occurred • Support by administration is also beneficial for reviews teams • Clinical governance or quality and safety staff provide expert guidance and oversight over multiple reviews • Some review panels include consumers and consumer representatives or seek feedback from patients and family members to add important depth • Investigators need clinical knowledge, an understanding of systems thinking and human factors, and to understand investigation methodologies and general principals • Dedicated recommendations teams support the review panel and refinement of recommendations Reviews, recommendations and implementation – Conducting reviews • Consulting with staff, teams • Consulting with patients and families Reviews, recommendations and implementation - Implementing recommendations • Timely implementation of recommendations is fundamental, to ensure those involved feel confident they can enact change	Health organisations and Wider System Influences - Organisational culture • Concerns about reputational damage resulting from PSI reviews Health organisations and Wider System Influences - Organisational structural processes • Resource intensive process of individual reviews for certain incident types (such as falls) causes frustration Selection of PSI Reviews - People involved • Training and experience of review team members is reportedly variable across jurisdictions, leading to challenges producing high quality reports with SMART recommendations and is influenced by staff turnover Reviews, recommendations and implementation - Developing recommendations • Time and workload of clinicians and review teams • Modification of recommendations by executive
Implementation process domain	Reviews, recommendations and implementation - Implementing recommendations • Accountability of management and executive to ensure implementation was also noted as being important • The recommendation implementation process can be tracked and monitored through that incident reporting system • The tracking of the recommendation implementation is also monitored by executive and safety and quality meetings, reporting on the progress and completion as appropriate. This process is variable. • Dashboards tracking completion of recommendation implementation regular meetings, and biannual reports may assist with accountability • Recommendation registers support the tracking and monitoring of the implementation of recommendations, particularly when accountability was tasked to a position, rather than an individual, to account for staff turnover	Reviews, recommendations and implementation - Implementing recommendations • Nurses in leadership roles (CNCs, NUMSs) are often tasked to implement recommendations in their teams, or areas, despite not always being aware of the context or antecedent of the recommendation • Limited shared learning about what has been successfully implemented elsewhere and why • Strong recommendations have the potential to be poorly implemented if there is limited support and expertise to support the process, and implementation becomes an ineffective tick box exercise, without achieving the intended change • Frustration reported that recommendations and their implementation strategies get modified through various bureaucratic • System-wide changes are particularly difficult to implement, as they involve cooperation and coordination of multiple interdivisional and interorganisational stakeholders, structures and approval processes within organisations, and what ends up being implemented is significantly different to what was intended by the review team, with limited accountability for effective implementation • There is not always a clear evaluation plan or process such as audits to ensure fidelity (implemented as intended). Weak recommendations that are wordy, not specific or measurable, are also challenging to follow up and implement

## Discussion

This study identified key challenges in the incident review process. Despite strong efforts to reduce a blame culture, resistance to reporting and blame persists in some organisations, while reducing over time in others. We observed: a lack of systematic consumer involvement in the PSI review process; limited independence within the PSI review process; shortcomings in shared learning across organisations and health services, including limited feedback on review findings, which impacts opportunities to improve; variability in the strength of recommendations resulting from PSI reviews, with few recommendations underpinned by systems thinking; poor development of implementation monitoring and evaluation strategies; limited governance and accountability for the achievement of change resulting from PSI reviews and the implementation of recommendations; and a need to re-balance the resources spent on in-depth PSI reviews vs. conducting more concise or thematic reviews and QI improvement efforts.

### Modifying the underlying blame culture

A blame-free approach to incident reporting, analysis, and learning has long been acknowledged as the most appropriate for effective prevention (99). The present analysis indicates that a persistent blame culture in some settings continues to be present. These findings suggest that further work is required to fully remove the blame culture from PSI, but that various barriers remain. The patient safety culture in a healthcare organisation underpins the reporting of incidents, the attribution of blame and embedded restorative just culture (100). It supports the identification of areas for improvement, evaluation of patient safety interventions, and benchmarking, through processes to effectively learn and improve from incidents (100). Hospitals with higher patient safety culture measures are associated with fewer adverse events (101). Similarly, a restorative just culture can enhance incident reporting, transparency, opportunities for learning, and psychological safety for staff involved in an incident (62, 102) [the second victim experience (95, 96)] by shifting the focus towards Safety-II which seeks to understand the everyday processes that work well (103). It is associated with increased performance resilience (104, 105), enhanced quality of review recommendations and lower rates of mortality (102, 106).

### Systematic consumer involvement in the PSI review process

This study identified wide variability in the involvement and engagement of patients and families, to understand their needs from PSI investigations, the questions they want asked, and hearing their perspectives on what happened. This cohort can provide valuable insight into the PSI, prioritise issues, and provide guidance on what they want a PSI review to achieve, in the hope to gain closure or regain trust in the healthcare organisation (65, 66, 70). Systematic engagement with this cohort is needed as we strive to improve the PSI response process and reduce patient risk of harm.

The findings also point to variability in the engagement of CRs within the PSI review process (70). Meaningful engagement of this group within the review process, particularly when it is more than a one-time engagement event (107), can provide alternative and broader perspectives (80). In general, the participants in this study recognised the value that patients, families, carers and CRs could add to PSI reviews, and were supportive of and working towards improving this process. There is an opportunity to make CR engagement more explicit and consistent by embedding it in organisational policy, providing structural training and resources (70). To maximise effectiveness, the provision of resources, tools, and training for staff and consumers is needed to facilitate meaningful engagement and input from patients, families and CRs (63).

### External contributors to the PSI review process

This study identified variability in the inclusion of external contributors to PSI review panels. Involving individuals from outside the organisation, whether a single external team member, an entirely external PSI review team, or an external investigation body, can help broaden perspectives, support accountability, impartiality and professionalism (108–110), and is common practice in other industries such as aviation (108, 109). However, it is important to critically reflect on the concept of “independence”. Safety science and human factors literature emphasise that all reviewers contribute different frameworks and assumptions, and no analysis is truly neutral (62). Rather than positioning external experts as impartial arbiters, our findings suggest the value of collaborative learning, where diverse forms of expertise, including lived experience and systems thinking, are brought together in ways that support mutual understanding and more holistic system-level insight.

Organisational barriers to engaging external contributors, however, exist. Some healthcare organisations may be reluctant to involve outsiders in review processes due to concerns about reputational risk, legal exposure, or loss of control over the narrative (111, 112). Acknowledging and addressing these tensions is crucial to fostering more open and participatory systems of learning, and lessons can be drawn from high-risk industries, such as aviation (113–115), where multidisciplinary collaboration supports both accountability and learning (110, 116). Health systems could benefit from adopting similarly transparent and restorative approaches. Integrating such approaches into PSI reviews in health can help shift the focus beyond local issues to broader structural and regulatory factors, while fostering rational, restorative and participatory forms of inquiry.

### The need to enhance collaborative learning

Collaborative learning is a critical component of effective incident response and harm prevention. This study revealed significant concerns about the limited and inconsistent dissemination of learning from PSI reviews. Rather than viewing learning as a unidirectional transfer of information, a collaborative learning approach recognises that meaningful change arises from shared reflections and dialogue across all levels of the system. This includes those directly or indirectly impacted by the incident or subsequent changes, such as local teams impacted by the implemented change or the original incident, as well as the wider organisation, and intra- and inter-state organisations (62). Poor dissemination of review findings, recommendations and implementation outcomes can result in replicated effort, missed opportunities for system-wide improvement and disengagement among staff (117). When learning is not visible, relational or participatory, it undermines the creditability of PSI review processes and trust in their capacity to drive improvement.

Collaborative learning can impact the visibility of action, and in turn the culture of reporting and general morale around the effectiveness of PSI review processes achieving change (108, 117). Sharing of review findings could also be used as a resource for future review teams to support the development of high quality recommendations and their implementation (117), but this is not currently done effectively. Further research involving the development, testing, and implementation of effective PSI review sharing processes is therefore recommended.

Embedded systems to disseminate and share findings [such as the National Patient Safety Alerts (118) in the UK, and the Safety Alert Broadcast system in NSW (57)], may support teams to enhance review outputs (117) as would the development of a repository of completed and ongoing incident reviews, contributing factors, recommendations and evidence-based implementation strategies (54). Feedback on the quality of the review and outputs from internal and external expert organisations also impacts the opportunities for review members to learn from incidents, may impact willingness to report PSIs (117, 119), and is an identified area for improvement. There is a clear need for a sector wide incident reporting and learning system that uses consistent review methods and processes, to allow analysis at the hospital, health service, state, and national level and to support benchmarking at each level (120).

This study also found that the sharing of review findings with patients and families remains limited, which can undermine trust and perceptions of transparency in the healthcare organisation (65, 66, 121). Collaborative learning emphasises that patients and families are not merely recipients of information, but essential partners in the learning process following harm. Meaningful engagement requires more than disclosing outcomes. It involves creating space for dialogue and recognising the knowledge of those affected (including investigators, clinicians and CRs) to support mutual understanding of what happened and why (66). These principles align with the idea of a learning health system, in which learning is continuous, transparent and grounded in partnerships with healthcare providers, patients and families (122). The Australian Open Disclosure framework (59) supports this principle and strongly recommends that PSI review reports should be shared with patients and families, in an accessible format and language, as well as written and face-to-face communication that includes acknowledgement of patient and family concerns, acknowledgement of and apology for the harm caused, identification of the contributing factors, and a clear explanation of the actions taken to prevent recurrence and monitor implemented changes (59). Strengthening this collaborative approach can contribute to healing, foster shared accountability and support system-wide learning.

### The challenges in developing effective and feasible recommendations

Recommendations are intended to improve processes and thus outcomes for healthcare consumers and staff (54). Poor quality or weak recommendations, however, remain a persistent issue (102), often with variable clarity of language used, impacting how recommendations are interpreted and how feasible and acceptable they are (54). Large numbers of weak recommendations being made increases the pressure across the healthcare system through the prioritisation and implementation of those improvements, leading to either poor or no-implementation or change (54). Failure to implement changes to prevent future harm can impact staff confidence in the PSI review and management process (108), as well as consumer confidence in the healthcare organisation or system (54).

Developing strong recommendations requires consideration of implementation science, systems thinking and humans factors (60), to ensure that system leverage points are targeted and that improvements use an evidence-based systems-thinking approach to reduce the likelihood of patient harm, and support staff and patients (123). Identification of contributing factors and recommendation development can be guided by established systems-based frameworks such as PreventiMaps (The Prevention Map) (124, 125). This approach considers systems-based contributing factors to support the development and implementation of effective recommendations (126). A critical feature of the PreventiMap approach is that it supports the development of a network of integrated recommendations that span all levels of the system in question, ensuring that the behaviour change desired at the sharp-end is facilitated through systemic change (126). Within this process, data sources used to guide the analysis of PSIs should incorporate observation of practice to ensure work-as-normal, and work-as-done is captured and considered within the analysis (127). This process also requires a skilled review panel to effectively consider and incorporate human factors and systems based thinking into the PSI review process (58).

Recommendation strength is typically assessed against the hierarchy of effectiveness (128), with *policies, guidelines and education* considered the least effective strategies, *standardisation and checklists* moderately effective, and *system-based change* considered the most effective tools to achieve change (129). For these to be effective, however, they need to be coupled with evidence-based implementation strategies (130). On their own, changes to policy or guidelines are considered weak recommendations, partly as unwarranted clinical variation in care is a persistent issue, and guideline adherence is a complex challenge, impacted by a multitude of factors (131–133). To increase the likelihood of effective implementation, recommendations should be high impact, and low effort, avoiding reliance on people's action by standardising or simplifying processes, introducing forcing functions or software changes, or architectural redesign (16). Frameworks such as PreventiMaps can support the development of recommendations that link changes across multiple levels, for example incorporating changes to policy along with education, and procedural changes, underpinned by evaluation and reporting to support adherence to the changes.

### The importance of effective implementation design and evaluation

Variable monitoring and evaluation of recommendation implementation was also highlighted in this analysis. The survey findings in particular identified that the vast majority of participants disagreed that recommendations were reliably implemented or lead to real improvements in patient safety. One recent review identified less than 7% of recommendations (from 4,579) as being system-focused, effective or strong (55). This relates to the methodologies used to investigate incidents (55), such as RCA, which have limited capacity to identify systemic contributory factors and produce strong recommendations (40, 45, 128), also supported by findings from this study.

Internationally, the implementation of weak recommendations (38, 54, 56) has limited impact on rates of PSIs and the risk of harm (38, 56). Improving the implementation of strong system-based recommendations, along with enhanced monitoring and evaluation of recommendation implementation is fundamental to achieve changes in care processes and patient outcomes. This fundamental finding is underpinned by complex barriers to effective implementation, requiring a multifaceted approach to improve implementation practices (38, 56). Enhanced incorporation of implementation science and quality improvement principles, including using small scale iterative tests like Plan-do-study-act cycles (134), may support planning for effective implementation and evaluation strategies, taking into account known barriers and facilitators within supportive implementation frameworks, thus increasing the likelihood of effective change being achieved (50–52).

### Governance and accountability for the implementation of recommendations

This study also identified limited systematic governance and accountability processes to ensure the implementation of recommendations is both accountable to a specific role and monitored in the short and long term. While monitoring in turn informs iterative changes to the implementation process to achieve effective change to patient risk and outcomes, this feedback loop is often weak or absent. These gaps reflect broader concerns in both national and international contexts. Limited governance structures are an international concern, limiting the processing of reported incidents, the review process, the implementation of recommendations and evaluation of impact of the PSI reviews (108). Although frameworks such as the Australian National Model Clinical Governance Framework explicitly hold health service executives and boards accountable not only for service delivery and financial performance but also for clinical governance (135, 136), this accountability is often not meaningfully enacted in practice. The tendency to prioritise operational and budgetary concerns can lead to a disconnect between the formal responsibilities of leadership and the day-to-day realities of implementing and learning from PSI reviews (137).

### Resourcing and proportionality of PSI reviews

This study identified concern around the majority of patient safety resources being dedicated to in-depth reviews of serious harm PSIs, to the detriment of reviewing lower harm incidents or near misses or conducting preventative QI activities. In safety science it is widely acknowledged that minor incidents provide the learning opportunities to prevent major incidents (138, 139). The response to PSIs should therefore be proportionate and consider the opportunity to learn from PSIs and prevent future risk (80). This study highlights the potential to reassess the criteria for conducting extensive in-depth reviews, and consideration of conducting fewer but more effective in-depth reviews, particularly of reoccurring incidents, that achieve improvements to practice and patient risk (80). More concise or thematic reviews and quality improvement should be undertaken as an alternative option to in-depth reviews, within a system of finite resources. This aligns with recent policy shifts such as the NHS PSIRF (18) and the updated London Protocol (58), which both advocate for a more flexible, proportionate and systems-oriented approach to incident response and learning. Patient safety reviews, particularly in-depth reviews, are often seen as the key strategy to minimise harm but are in fact one of many tactics that can be employed by a health system. Other tactics include identifying, monitoring and managing clinical risk and ensuring a culture of learning and improvement (140).

### Policy implications

Despite decades of research and efforts to improve the response to PSIs, findings from this study indicate limited impact and improvement. This is highlighted by the attitudinal survey findings that identified only half of the participant cohort agreed that recommendations were accepted, appropriately targeted, consider what went well as well as what went wrong, and are achievable, that learning from PSIs prevents recurrence, and that investigators have access to the rights tools, support, skills and knowledge to conduct quality reviews and achieve change.

Findings from this study, in conjunction with other arms of this project (20, 70, 71) will inform the development of Best Practice Principles for learning and improving from PSIs, and a codesign process to develop and test system-wide improvements to PSI review processes and implementation. The Best Practice Principles and co-design process will be deliberately wide in scope as the incident review process is complex and has many influences across the health system and beyond. The Best Practice Principles will draw upon relevant frameworks and systems thinking from safety science, policy frameworks, implementation science, organisational culture and learning health systems to ensure the approach is both evidence-informed and context-sensitive. Isolated changes in one part of the process, for example, changing the composition and expertise of the investigation team, is unlikely to have an impact on the overall effectiveness of the incident review process to reduce patient harm. A system-wide end-to-end re-thinking and re-engineering of the process is likely to be required.

Re-engineering patient safety practices to incorporate these Principles will require system and behaviour change supported by and reflected in policy change to implement a blame-free systems thinking-based approach to PSIs with an emphasis on systemic change and improvement. Proposed improvements are outlined in Table 3. The feasibility and acceptability of such changes will be trialled in the next codesign and feasibility phases of the project.

**Table 3:** Proposed changes to re-engineer the PSI review process.

Proposed re-engineering changes
Organisations should understand their capacity to undertake robust PSI reviews (157)
Consideration of patient, family and staff needs incorporating restorative justice principles (15, 17, 65, 66, 107)
Proportionality of response (103, 158) and opportunities to learn from incidents (48, 103) within the PSI review criteria
Inclusion of external review members, consumers and human factors and system perspective specialists in PSI review panels (58)
Greater use of observation of real work (128)
Thematic analysis (159) and concise review to inform the analysis of contributing factors for PSIs (126, 127)
Systematic application of human factors and systems thinking to identify contributing factors and develop evidence-based and effective recommendations (23, 126)
Testing and evaluation of recommendations prior to implementation (52, 134)
Explicit reporting of evidenced based improvements, data sources, consumer input, conflict of bias, implementation plans and plain language summaries and footnotes within review documentation; continual monitoring and evaluation of implementation of recommendations to enhance accountability (46, 105, 160)
The transparent sharing of findings with families and broader health system (117, 161, 162)
System wide analysis and review of key patient safety risks, themes and contributing factors within and between health services. (128, 163–165)

### Strengths and limitations

This study includes a large analysis of Australian and international patient safety experts and healthcare professionals’ perspectives regarding PSI reviews. Rigour was enhanced through the use of triangulation of perspectives from participants across a variety of backgrounds, and settings, as well as survey findings, and the utilisation of multiple coders during the data analysis. A key strength was the inclusion of perspectives from health services that account for 78% of Australia's public hospital admissions, but the perspectives of HCPs from other public health and private services were not represented in the data, limiting to some degree, how generalisable the findings are across Australia. This study primarily reflects the perspectives of participants working in quality and safety roles, senior clinicians and safety professionals, many of whom are embedded within the healthcare hierarchies and institutional models of expertise. As such, the findings may not fully capture the experiences or frontline clinical staff or those who have been the subject of investigations and consumers, whose insights are critical to understanding the broader impact and effectiveness of PSI review processes. Future research should seek to include these voices to support more inclusive system learning. The research utilised a concurrent MMR approach. Had the research been conducted using a sequential MMR design, the survey findings may have informed the interview questions allowing for more nuanced investigation of participant perceptions and experiences.

## Conclusion

PSI reviews are failing to support effective prevention of patient safety incidents. This study has provided insights into current practices and perceptions of the PSI review process. Key findings will inform codesigned improvements to better support review teams to learn from incidents and prevent future patient harm: PSI review panels should systematically incorporate input from external experts as well as CRs; continuous efforts to reinforce a just restorative and blame-free culture is fundamental to support incident reporting, and development and implementation of effective systems-focused recommendations; effective recommendation development requires consideration of systems thinking and humans factors; monitoring and evaluation of recommendation implementation is a fundamental mechanism to enhance accountability and achieve change; and finally, broad and transparent sharing of review findings will support the development of higher quality review and recommendations These strategies should not be implemented in isolation. The complexity of healthcare systems demands a coordinated, whole-of-system re-engineering of investigation processes, shifting from compliance-based reviews towards strategic, relational, restorative and sustainable approaches that support continuous improvement.

## Data Availability

The datasets presented in this article are not readily available because the data supporting the conclusions made in this paper may be available on request from the corresponding author subject to ethics and confidentiality requirements. Requests to access the datasets should be directed to Peter Hibbert peter.hibbert@mq.edu.au.
