# Supplementary material for: Decades of failure to prevent harm to patients—where are we going wrong? A mixed methods study of the perspectives of health services staff across Australia and internationally
Source: Front Health Serv. 2025 Sep 2;5:1645575. doi: 10.3389/frhs.2025.1645575 (PMC12436320; doi:10.3389/frhs.2025.1645575)
Supplement: Supplementary file 1 [file Datasheet1.docx]

**Supplementary file 1**

**Good Reporting of A Mixed Methods Study (GRAMMS)**

Source: O'Cathain, A, Murphy, E and Nicholl, J. "The quality of mixed methods studies in health services research." Journal of Health Services Research & Policy 13.2 (2008): 92-98.

| GRAMMs criteria | Page |
| --- | --- |
| Describe the justification for using a mixed methods approach to the research question | 4 |
| Describe the design in terms of the purpose, priority and sequence of methods | 4 |
| Describe each method in terms of sampling, data collection and analysis | 4-6 |
| Describe where integration has occurred, how it has occurred and who has participated in it | 5-8 |
| Describe any limitation of one method associated with the presence of the other method | 21-22 |
| Describe any insights gained from mixing or integrating methods | 5-8 and 16-22 |
